# Optimal Control Theory for Personalized Therapeutic Regimens in Oncology: Background, History, Challenges, and Opportunities

**DOI:** 10.3390/jcm9051314

**Published:** 2020-05-02

**Authors:** Angela M. Jarrett, Danial Faghihi, David A. Hormuth, Ernesto A. B. F. Lima, John Virostko, George Biros, Debra Patt, Thomas E. Yankeelov

**Affiliations:** 1 Oden Institute for Computational Engineering and Sciences, The University of Texas at Austin, Austin, TX 78712, USA; ajarrett@utexas.edu (A.M.J.); david.hormuth@austin.utexas.edu (D.A.H.); lima@ices.utexas.edu (E.A.B.F.L.); biros@oden.utexas.edu (G.B.); 2 Livestrong Cancer Institutes, The University of Texas at Austin, Austin, TX 78712, USA; jack.virostko@austin.utexas.edu; 3 Department of Mechanical and Aerospace Engineering, The University at Buffalo, Buffalo, NY 14260, USA; danialfa@buffalo.edu; 4 Department of Diagnostic Medicine, The University of Texas at Austin, Austin, TX 78712, USA; 5 Department of Oncology, The University of Texas at Austin, Austin, TX 78712, USA; 6 Department of Mechanical Engineering, The University of Texas at Austin, Austin, TX 78712, USA; 7 Texas Oncology, Austin, TX 78731, USA; Debra.Patt@usoncology.com; 8 Department of Biomedical Engineering, The University of Texas at Austin, Austin, TX 78712, USA

**Keywords:** mathematical model, cancer treatment, predicting response, optimizing response

## Abstract

Optimal control theory is branch of mathematics that aims to optimize a solution to a dynamical system. While the concept of using optimal control theory to improve treatment regimens in oncology is not novel, many of the early applications of this mathematical technique were not designed to work with routinely available data or produce results that can eventually be translated to the clinical setting. The purpose of this review is to discuss clinically relevant considerations for formulating and solving optimal control problems for treating cancer patients. Our review focuses on two of the most widely used cancer treatments, radiation therapy and systemic therapy, as they naturally lend themselves to optimal control theory as a means to personalize therapeutic plans in a rigorous fashion. To provide context for optimal control theory to address either of these two modalities, we first discuss the major limitations and difficulties oncologists face when considering alternate regimens for their patients. We then provide a brief introduction to optimal control theory before formulating the optimal control problem in the context of radiation and systemic therapy. We also summarize examples from the literature that illustrate these concepts. Finally, we present both challenges and opportunities for dramatically improving patient outcomes *via* the integration of clinically relevant, patient-specific, mathematical models and optimal control theory.

## 1. Introduction

Treatment of cancer has progressed considerably since the first radiation treatments in the late 1800s [1] and the early chemotherapies derived from mustard gas in the 1920–40s [2]. Today, therapeutic regimens can include radiation therapy, cytotoxic approaches, targeted therapies, and/or immunotherapy in addition to surgical resection. While the number and types of therapies available for cancer treatment have dramatically expanded over the past century, the dosing and timing of administration is still relatively imprecise. Standard-of-care treatment regimens are based on the results of expensive and time-consuming clinical trials that seek to first determine the maximum tolerated dose (Phase I), and then the expected efficacy for an “average” patient (Phases II and III). As it is impossible to systematically evaluate all possible dosing schemes within the clinical trial system, it is largely unknown what are the “best” ways to schedule radiation and systemic therapies. The problem is further compounded when multiple therapies are being prescribed, as is done in the majority of cancer cases. For combination therapies, medications can be administered simultaneously or sequentially, without complete knowledge of the potential synergistic or antagonistic relationship these treatments can exhibit based on their order and timing of administration. In fact, several preclinical studies have shown that the order and timing of certain targeted and cytotoxic therapies may not be optimal [3,4], and clinically there is evidence for certain combinations of drugs for which the sequence of their administration can significantly affect their efficacies and toxicities [5]. Thus, oncology is in desperate need of a practical, clinically relevant, logical framework that allows the investigator to *a priori* compute the optimal therapeutic regimen on a patient-specific basis.

Biological process-based mathematical models, when initialized and calibrated with patient-specific data, may dramatically enhance the efficacy of current therapies through the methods of optimal control theory (OCT). In OCT, models can be specialized for individual patients to make personalized predictions that are “actionable” in the clinical setting. Compared to the clinical trial system, the use of mathematical models permits the systematic, *in silico* study of numerous possible formulations of dosing, timing, and combinations of therapies. Furthermore, with formal application of OCT, the costs of therapy (including toxicity, efficiency, psychological, quality of life, as well as economic considerations) can be weighed against the effectiveness of the regimen, so that an optimal regimen can be defined for not only subgroups of cancer patients but also for individual patients.

In this review, we first summarize the historical approaches for determining therapeutic regimens in medical and radiation oncology. Then, we introduce the mathematical underpinnings of OCT and illustrate cases of the technique being used with mathematical models of tumor growth and treatment response. Next, we discuss the current challenges preventing fundamental progress in using OCT and mathematical models to guide therapeutic decisions—including the lack of readily accessible data to adequately characterize patient-specific characteristics and the lack of practical theoretical formalisms to compute the optimal regimen for an individual patient. Lastly, we identify several exciting opportunities for future optimization of cancer treatment, such as quantitative imaging data to characterize the tumors of individual patients, multiscale modeling to incorporate additional layers of patient-specific data into the planning of therapy regimens, and the prospect of optimizing combination therapies.

## 2. Current Approaches for Establishing Therapeutic Regimens

Many standard-of-care approaches to treating cancer consist of both of chemo- and/or radiation therapy. Therefore, we focus on these two fundamental treatment modalities in cancer but note that immune and targeted therapies share similar opportunities and challenges for determining optimal therapeutic regimens. 

### 2.1. Systemic Therapy

**Figure 1:**
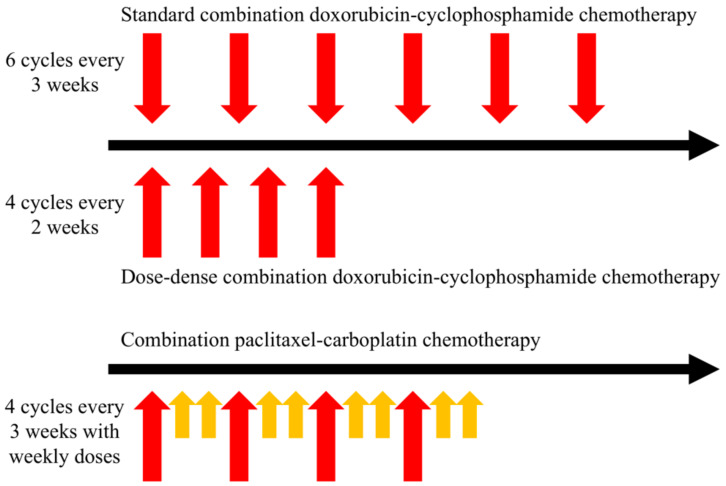
Comparison of three neoadjuvant regimens of chemotherapy for triple-negative breast cancer. Red arrows represent the first dose of every cycle, and yellow arrows represent doses during the course of a cycle. The dose-dense combination doxorubicin-cyclophosphamide therapy regimen consists of fewer cycles over a shorter period of time with the same dosage compared to the standard regimen—where both drugs are given for every dose. While patients are more likely to experience side effects during the dose-dense regimen (with shorter recovery periods between cycles), the treatment is completed in half the time. For the combination paclitaxel-carboplatin regimen, carboplatin is administered at the beginning of each cycle (4 doses), and paclitaxel is given weekly (12 doses). Without a framework to evaluate these regimens against each other on a patient-specific basis, there is no way to know which regimen (or an alternative regimen) may be more beneficial for treatment. Given clinically relevant models of tumor response to therapy, optimal control theory (OCT) may be employed to systematically investigate numerous regimens *in silico* to identify optimal treatment approaches on an individual patient basis.

Chemotherapy is normally administered (individually or in combination with other drugs) over units of time termed “cycles”, which are regular intervals over the entire treatment period. These cycles normally span days to weeks depending on the treatment plan, where the amount of time between cycles is thought of as a “recovery period” for the patient and their normal, healthy cells. Figure 1 illustrates three common examples of regimens used for two types of neoadjuvant chemotherapy (i.e., therapy before surgery) in breast cancer. Note that these regimens can vary in their frequency, duration, and dosage across regimens and even for the same therapy. Additionally, in the standard-of-care setting, this treatment paradigm may be modified depending upon each patient’s individual response as well, with consideration of their overall health and quality of life. Oncologists choose treatments using decision tree algorithms that have some specificity. The gold standard for these algorithms is the National Comprehensive Cancer Network guidelines (www.nccn.org) based on tumor size, degree of spread, and molecular characteristics. Dosing of therapies requires the careful balance of maximizing the anti-tumor effect while simultaneously limiting toxicity to acceptable levels, for which OCT may provide valuable insights.

Therapeutic efficacy refers to the potential beneficial change due to a drug. Treatment efficacy is very difficult to evaluate, as there can be a significant time lag between drug administration and objective tumor response. Further complicating the evaluation of therapeutic efficacy is that direct measurement of the concentration of drug reaching a tumor is difficult to assess. While there are some imaging-based measures (typically based on radiolabeled drugs; see, for example, Reference [6]), they are not widely available or utilized. The most common method of assessing drug delivery is *via* pharmacokinetic analyses of peripheral blood, which do not necessarily reflect the amount of drug in tumors that often have limited and heterogeneous perfusion. Toxicity (potentially damaging or poisonous effects) is commonly reduced by decreasing or skipping doses, but this comes at the expense of reduced efficacy [7,8]. Often during systemic therapies, patients will be given additional, supportive medications in an attempt to alleviate side-effects and help the patient recover more quickly from the therapy. This strategy is motivated by the fact that without alternative regimens to compare to, oncologists strive to have patients on the standard-of-care therapy schedules, receive their full doses, and complete a full course of the therapy.

In an attempt to balance therapeutic efficacy and toxicity, the total dose of a particular chemotherapy is typically determined by the body surface area (BSA) of the patient, a practice established over 60 years ago to extrapolate dosage between species [9]. However, BSA is not measured directly but rather estimated from height and weight using one of a series of accepted regression equations—the results of which can vary widely [10]. Furthermore, a number of factors besides BSA have been shown to influence drug distribution, including hepatic and renal function, body composition, enzyme activity, drug resistance, gender, age, and prior/concomitant medication [11]. Given these shortcomings, the fact that BSA-based dosing fails to reduce variability in drug efficacy [11] is unsurprising. Alternatives to BSA-based dosing strategies rely primarily on pharmacokinetic modeling due to the observed relationship between plasma dynamics and drug toxicity that exists for many anticancer drugs [12]. Population-based pharmacokinetic models can be combined with data from an individual patient using mathematical modeling to guide drug dosing [13]. These models can also incorporate patient-specific factors known to affect drug retention and clearance, such as renal function or enzyme variants [13]. Oncologists also use simple mathematical models to provide estimates of risk reduction conveyed by embarking upon a given regimen of treatment using various types of data, like genomic profiling [14,15] and actuarial data [16]. However, these modeling methods are quite limited and are used variably throughout the standard of care. Thus, the selection of optimized regimens, feasibly administered in the clinical setting, would be invaluable toward this effort.

In summary, defining therapeutic regimens for chemotherapy using methods that are unable to precisely measure either therapeutic toxicity on healthy cells or therapeutic efficacy on cancer cells, is an extremely difficult problem. While each treatment plan is developed considering the balance between efficacy and toxicity, it is based on historical data and physician skill; in particular, there is currently no way to rigorously determine this balance for optimal patient outcome. Furthermore, these methods do not consider how drug-induced resistance may inhibit treatments. Beyond the need to develop more accurate methods for quantifying the toxicity and efficacy of therapeutics, the systematic generation of alternative dosage strategies for individual patients is also necessary. Integrating OCT with accurate mathematical models of tumor growth and treatment response may be able to identify alternative regimens.

### 2.2. Radiation Therapy

Similar challenges exist in the development of effective dosing regimens for radiation therapy as those just discussed for systemic therapy. Again, a fundamental challenge is to develop therapeutic regimens that maximize tumor cell death while simultaneously minimizing toxicity to normal tissues. Radiation therapy was initially given as large single doses in an attempt to eradicate tumors [17]. However, these large doses frequently resulted in serious complications, motivating investigation into alternative approaches. 

Currently, multiple fractions of a low dose (~2 Gy) is typically utilized as it maximizes the therapeutic efficacy while largely sparing healthy tissue. In some disease locations, increasing the dose much beyond 2 Gy results in a disproportionate increase in damage to healthy tissue relative to tumor tissue, thereby decreasing the therapeutic ratio (i.e., the ratio of the maximum tolerated dose to the minimal effective dose). However, 2 Gy alone is insufficient to result in tumor control, so it is typically given over many fractions to reach a cumulative dose ranging from 50 to 80 Gy [18,19,20,21]. Delivering radiation therapy over several fractions (not unlike chemotherapy cycles) allows healthy tissue to recover while causing damage to cancer cells that were previously not actively proliferating, grew between fractions, and/or have become less hypoxic (oxygen deprived/starved) [22]. With the advent of image-guided radiation therapy, alternative regimens are being considered that can deliver more localized doses of radiation. For example, hypofractionation (i.e., fractionated therapy that delivers greater than 2 Gy per fraction over fewer total fractions) may result in improved tumor control and shorter overall treatment plans [23,24,25]. Similarly, dose escalation (increasing of the total cumulative dose to the tumor while maintaining the same dose delivered to normal tissue) is also possible and may result in improved tolerance to the treatment and improved tumor control [26,27]. 

Another consideration for optimizing radiation therapy regimens is how to address the heterogeneity in radiosensitivity between patients and even within an individual tumor. In particular, differences in hypoxia across a tumor [28] can have a substantial influence on radiation sensitivity requiring a larger dose compared to normoxic tissue (i.e., tissue with normal oxygen levels). Briefly, during radiation therapy, highly reactive free radicals are produced that ultimately lead to tissue damage. In well-oxygenated tissue, these free radicals react quickly with oxygen and eventually form a stable chemical composition, resulting in permanent damage. In poorly oxygenated tissue, there is insufficient oxygen to react with the produced free radicals, and the chemical composition of the target tissue returns to its pre-irradiation form. It has been suggested that doses increased by a factor of up to three are needed to achieve a similar level of damage [29]. Ongoing developments in the noninvasive assessment of tumor hypoxia [30,31,32,33] are being explored to guide dose boosting to hypoxic regions to improve tumor control [33,34]. As with chemotherapy, more studies are needed to evaluate alternative dose and fractionation regimens to determine the overall clinical therapeutic benefit. 

## 3. Fundamentals of OCT

OCT is a mathematical and computational method that is applicable to a wide variety of scientific and engineering applications ranging from economics [35,36] to aeronautics [37,38]. This theory was initially developed for dynamical systems where the evolution of the variables may be directed by external controls. Optimal control aims to determine the specific factors controlling a system (e.g., a dosing regimen for treating cancer) so that a specific criterion can be achieved (e.g., maximizing tumor control while minimizing side effects) [39,40]. While a detailed discussion of the mathematical theory behind optimal control methods is beyond the scope of this paper, we will provide a brief introduction to the theory with an emphasis on the concepts important for its application to cancer treatment. The reader interested in the mathematical details is encouraged to study the Supplementary Materials and the references provided throughout the following sections, as well as other cancer-relevant examples of optimal control that can be found in References [41,42,43,44]. 

Consider a dynamical system describing tumor growth evolving in time, *t*, where the volume, cellularity, or some other tumor measure is changing with time, *T*(*t*), written as an ordinary differential equation (ODE):

(1) $$\frac{dT}{dt}=f(T(t),\;z(t),\;t),$$

where *f* can be any type of tumor growth model (e.g., Gompertzian or logistic growth [45]) and is subject to (and therefore potentially controllable by) chemotherapy or radiation treatment, *z*(*t*). The basic objective for optimizing therapy is to identify a dosage plan, *z*(*t*), such that *T*(*t*) is minimized at the final time *t* = *t_f_*. Therefore, in the vocabulary of optimal control theory, *T*(*t*) denotes the state variable that describes the behavior of the dynamic system, and *z*(*t*) is the control variable that affects the dynamics of the state by acting upon it. To formulate the optimal control problem, we must define the objective functional that we wish to minimize that accounts for the dynamics between the tumor growth and therapy.

### 3.1. What is an Objective Function?

For the optimal control problem, an objective functional, *J*, is defined, where the aim is to adjust the control variable to minimize (or maximize) *J*. The objective functional consists of scalar terms Φ and *L* referred to as the endpoint cost and integrand, respectively. The endpoint cost refers to the result, considering the final result of the tumor and its treatment at the end of the therapy, where *t_f_* denotes the final time. The integrand accounts for the evolution of the tumor over the entire treatment period. The objective functional is generally written as:

(2) $$J=\Phi \left[u\left(t_{0}\right),t_{0},z\left(t_{f}\right),t_{f}\right]+\overset{t_{f}}{\underset{t_{0}}{\int }}L\left(u\left(t_{0}\right),z\left(t_{f}\right),t\right)dt,$$

where *t*_0_ is the initial time for the therapy, and *u*(*t*) is a vector of state variables that may be considered for optimization. Similarly, the vector *z*(*t*) may represent multiple types of therapies (e.g., chemo-, targeted-, or immune-therapies). As there may be several state and/or control variables, minimizing (or maximizing) *J* will require balancing the desired goal (e.g., maximizing tumor reduction) with the cost caused by the control (e.g., minimizing therapeutic toxicity). For the example described by Equation (1), where we want to minimize *T*(*t_f_*) and if we are ignoring the side-effects (or cost) of treatment, the cost functional *J* in Equation (2) can be rewritten with Φ = 0 and *L* = *u*(*t*) = *T*(*t*). However, one could imagine reframing this problem to consider radiation damage to healthy tissue, where our model system would include a governing equation for the dynamics of normal cells affected by therapy.

### 3.2. Why and How Should Constraints be Applied to the Cost Function?

Depending on the physical system Equation (1) is modeling, the optimal control problem may need to include constraints imposed on the state and control variables, such as:

*C*(*u*(*t*),*z*(*t*),*t*) = *a*, (3)

*S*(*u*(*t*),*z*(*t*),*t*) ≤ *b,* (4)

where *C* and *S* denote arbitrary, continuous, and possibly nonlinear functions. In the example outlined in the previous paragraph, *C* could describe the total dose that is administered, e.g., $\int_{t_{0}}^{t_{f}}z(t)dt\;\leq \;z_{\max},$ where *z*_max_ is a bound on the total drug exposure (see Equation (12) in Section 5.1 for a specific example limiting the total dose). Similarly, *S* could describe the dosing frequency. For example, if we define the total amount of time for the therapeutic regimen as *t*_tot_, then a limit on the frequency of doses can be defined by *f* ≤ *t_tot_*/*N*, where *N* is the maximum number of doses per treatment period. In this case, the control can be defined using the Heaviside function, *H*, as *z*(*t*) = *z_0_*$\overset{N}{\underset{k=0}{\sum }}H(t)$, where $z_{0}$ is the concentration of the drug administered and

(5) $$H(t)=\left\{\begin{array}{l} 0,\;\;\;\mathrm{otherwise}\\ {1,\;\;2t}_{k}+f\;\geq \;t\;\geq {\;2t}_{k} \end{array}\;\right.$$

for each timepoint *t_k_* = *kf*. In practice, the total dose that can be administered is fixed, but an optimal control problem can be allowed to vary individual doses over time. Additionally, the timing of doses has practical limitations, e.g., treatment is rarely given in the middle of the night, and the duration of each individual dose in a regimen may be constrained. Other forms of state and control constraints may need to be considered, including prescribed final conditions (i.e., restricting the total duration of the full course of treatment), see References [39,40] for a larger set of possible constraints.

To reiterate, the principal goal of an optimal control problem is to identify a set of necessary and sufficient conditions that the optimal control, *z*(*t*), and the corresponding state, *T*(*t*), must satisfy. The optimal control of systems governed by ODEs are commonly used [46,47]; however, the challenge of generating personalized cancer treatment regimens may require accounting for spatial components as well [48,49,50,51,52,53,54] and, therefore, necessitate system dynamics that are governed by spatially resolved models, such as partial differential equations (PDEs). For systems described by PDEs, obtaining the solution of the optimal control problem requires variational formulations of PDEs and appropriate solution spaces with respect to the state and control variables [55,56,57,58].

### 3.3. How do We Solve Optimal Control Systems?

In general, closed form solutions for optimal control problems do not exist, and therefore numerical methods are required to estimate their solution. The numerical solutions of optimal control problems can be categorized into indirect and direct methods. Indirect methods seek to find an alternate formulation of the problem before numerically solving. There are two different major approaches for achieving this goal: The Dynamic Programming Principle [59] and the Pontryagin Minimum (or Maximum) Principle (PMP) [60]. The Dynamic Programming principle transforms the optimization problem (i.e., Equations (1)–(4)) into a differential equation, known as the Hamilton–Jacobi–Bellman equation [40]. We describe in detail the PMP indirect method along with an example in the Supplementary Materials. For additional details on indirect methods, the interested reader is referred to Reference [61]. The primary practical drawback of indirect methods is the requirement of deriving analytic expressions for the optimality conditions. This requirement may be cumbersome for problems involving nonlinear objective functions or constraints. In contrast, direct methods are based on discretizing in time the state and control variables so that an analytical expression for the optimality condition expression is not required. The optimal control problem can then be solved using an iterative, nonlinear optimization algorithm [62] (such as steepest decent, Newton, or Levenberg–Marquardt methods) to identify an approximate control and state solution. However, the accuracy of the solution depends on the iterative method and discretization [63]. We note that the choice of the numerical solution method depends on the optimal control formulation, including the type of underlying model and the form of the cost functional along with the imposed constraints.

## 4. Formulating the Optimal Control Problem for Cancer Therapy

### 4.1. Overview of Mathematical Models of Tumor Growth for Optimization

A common goal for applications of OCT to cancer treatment is to minimize the tumor size (the state variable being volume, total cellularity, etc.) at a final time point. To be clinically relevant, it is critical that the state equation is capable of accurately predicting tumor growth and the interaction of the treatment with malignant and healthy tissue. In this regard, employing mathematical models that are built on the underlying biological and physical mechanisms of tumor growth and treatment response is fundamental for determining a rigorous optimal treatment plan [64,65]. The development of tumor models that characterize the relevant biophysical phenomena determining tumor initiation, growth, invasion, and metastasis, as well as the effect of therapy, is the subject of active investigation. Current mathematical models of tumor growth and treatment are either discrete (cell-based), continuum, or hybrid discrete–continuum models [45]. In this review, we will focus on continuum models.

The continuum approach takes into account the global tumor (or cell population) behavior and has been utilized to model angiogenesis [49,66], nutrient evolution and consumption by tumor cells [67,68], mechanical [50,69,70,71], and chemical [72,73,74] cues that affect tumor cells, as well as the effects of radiotherapy [75] and chemotherapy [48,76]. Of the continuum-based approaches, ODE models of tumor development are the most widely used for optimizing cancer treatment plans. However, ODEs are (of course) unable to capture the spatial heterogeneity of a growing tumor as they can only simulate the time evolution of one or more scalar quantities (e.g., tumor size, cell number, and temporally varying therapies) that describe an average over the spatial domain. In contrast, PDE models enable the simulation of both the temporal and spatial variations in these quantities. In particular, recent advances in *in vivo* imaging techniques [77] have provided quantitative data to initialize and calibrate model parameters, thereby enabling the use of PDE models of tumor growth to compute optimized treatment planning of greater clinical relevance [78,79].

While this review is focused primarily on continuous models, we note that there is much recent work in developing and applying agent-based models to the cancer treatment problem, which track and update individual cell dynamics using a set of rules. We refer the interested reader to Reference [80] for a recent review of agent-based modeling in cancer.

### 4.2. Qualitative Discussion of the Cost Functional and Control Constraints

The first control problems formulated to optimize chemotherapy were established almost 50 years ago by Swan [81] and Bahrami and Kim [82]. Since those initial efforts, several investigators have attempted to solve the dose scheduling problem as a constrained optimization problem with objectives that minimize the number of cancer cells or tumor volume at a final time point [83,84,85,86,87,88]. While the main objective of the optimal control problem is to minimize the cancer at a final time, *T*(t_f_), other criteria should be carefully built into the cost functional—usually through a weighted sum of a number of functions based on their level of importance. As indicated above in Section 2.1 and Section 2.2, both chemo- and radiation therapy may damage normal tissue, with potentially irreversible and life-threatening consequences. Such competition between the efficacy and toxicity effects of drugs demands careful design of therapeutic regimens to maximize the reduction of tumor burden while simultaneously minimizing toxicity and damage to healthy tissue [78,89]. Additionally, financial limitations and logistical restrictions of the treatment may need to be considered in formulating a clinically relevant optimal control problem. Therefore, optimal dose treatment is a multi-criteria optimization problem, in which advancing performance for one decision criterion may decrease the performance of another [78]. Having multiple criteria complicates the process in that neither condition should dominate or be considered superior to the other, and the optimal control solution represents the optimal tradeoffs among all decision criteria (known as the Pareto optimal) [90]. Additionally, a practical implementation of OCT to cancer must be derived according to the decision criteria and restrictions faced by clinicians.

**Figure 2:**
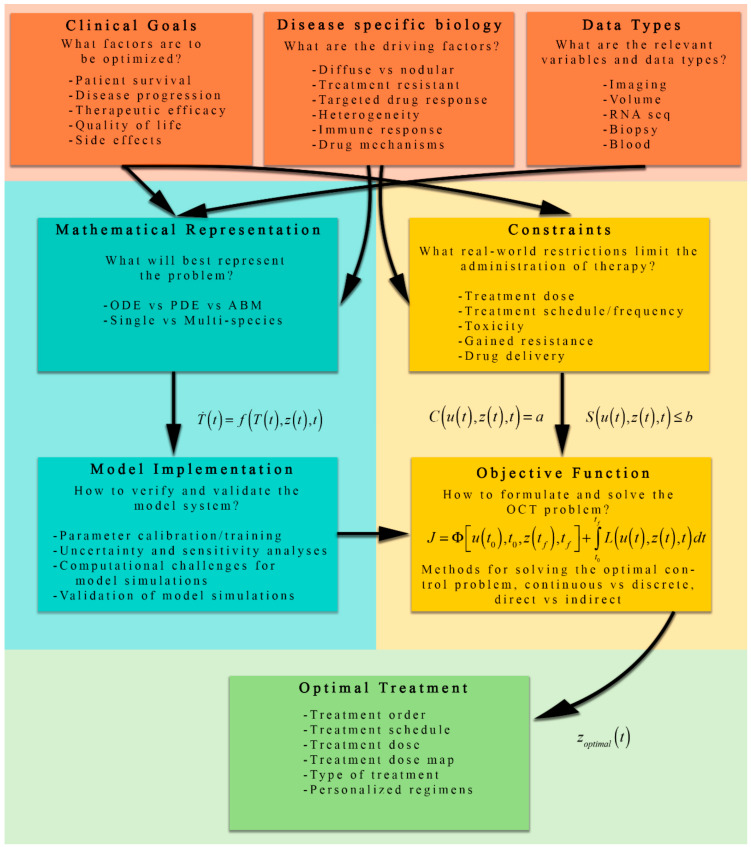
A schematic of the process for formulating OCT problems for cancer therapeutic regimens. The orange boxes represent key factors that influence the mathematical representation (teal boxes), optimal control implementation (yellow boxes), and eventually the optimally selected treatment (green box). Clinical goals, disease-specific biology, and available data influence both the development of the mathematical representation (i.e., the model) and the identification of constraints critical to using OCT to solve the problem. The mathematical representation of the dynamic system is then implemented numerically. The OCT structure uses the constraints (derived from real-world considerations) and the implemented model to systematically evaluate the objective function to determine the optimal treatment.

Figure 2 presents an overview of the process by which OCT is applied to cancer, along with a number of the considerations specific to each individual component of such a study. Notice that the formulation of the optimal control problem begins with three driving (or constraining) factors: clinical goals, disease-specific biology, and the available data. Each of these factors have direct consequences on the choices for the mathematical representation of the biological phenomena (Equation (1) above) and the implementation of the OCT problem (Equations (2)–(4)). The figure highlights several key questions to consider, such as “what factors are to be optimized?” and “how to verify and validate the model system?”, moving through each step of the process. After the formulation of the model system and objective function with constraints, the optimal control problem can be solved, and the optimal treatment regimen/plan/schedule/dose may be determined.

## 5. Applications of OCT in Cancer Therapy

OCT has been applied to tumor growth and response scenarios considering chemotherapy (mono- and combined therapy), targeted treatments, immunotherapy, radiotherapy, and combinations of these approaches [43,81,91,92,93,94,95,96,97,98,99,100,101,102,103,104,105,106,107,108,109,110,111,112]. Here, we will focus on examples of OCT applied to chemotherapy and radiotherapy that demonstrate the importance of selecting an appropriate model system and objective function. The details of a complete OCT example outlining the steps for using PMP are presented in the Supplementary Materials. In this section, we present summaries of several illustrative examples designed to build up the reader’s intuition for applying OCT to common problems in oncology.

### 5.1. Examples of OCT Applied to Chemotherapy

As noted above in Section 4.2, the first paper applying OCT to chemotherapy was the work by Swan et al. [81,113], where the authors combined the effects of multiple drugs into a single control variable for the treatment of multiple myeloma. The tumor cell growth is assumed to be Gompertzian, and the number of cells decrease due to the action of the therapy. The corresponding ODE model is:

(6) $$\frac{dT(t)}{dt}=-rT(t)ln(T(t)/K)\;-\;\alpha z(t)T(t)/(\gamma +z(t))$$

where *T*(*t*) is the number of tumor cells at time *t*, *K* is the carrying capacity, *z*(*t*) is the drug dose, *r* is the proliferation rate, *α* is the maximum death rate by the drug, and *γ* is the dose at which the death rate is half of *α*. The objective of the optimal control problem presented in Reference [81] is to reduce the size of *T*(*t*) to *T*(*t_f_*) = *T*_low_, where *T*_low_ and *t_f_* are a target tumor size and the final time, respectively, while minimizing *z*(*t*) by penalizing the use of the drug (a similar formulation was used by Bahrami and Kim [82]). The form of the resulting objective functional is:

(7) $$J\left(z\right)=\int_{0}^{t_{f}}z\left(t\right)dt.$$

In Reference [81], the optimal treatment plan is compared with patient data going through the standard treatment protocol. The standard protocol was 11 cycles of 1 dose every four weeks, resulting in the tumor reducing to *T*_low_. The resulting optimal treatment plan that satisfies the objective function, Equation (7), is to start with less than 1/10 of the standard dose, and gradually increase to half of the standard dose. With the continuous drug delivery defined by the optimal control solution, the treatment is able to reduce the tumor to *T*_low_ seven times faster than the standard protocol, with 1/40 of the standard accumulated dose.

Similar to Equation (7), a commonly used cost function to optimize therapeutic regimens based on an ODE models is:

(8) $$J\left(z\right)=T\left(t_{f}\right)+W\int_{0}^{t_{f}}\left(z{\left(t\right)}^{2}\right)dt,$$

where *W* is a subjective weight factor (i.e., variable of choice) that balances the relative importance of the two terms in the cost function (see, e.g., References [102,110,114]). The choice of *W* is problem- and drug-dependent. In Equation (8), *W* might be increased to lower the cumulative dose delivered in an effort to reduce the potential toxicity of the drug. However, if the main goal is to decrease the tumor at the end of the treatment, with little regard to the cumulative dose, *W* can be reduced. This weight is selected such that the desired feature of the optimal solution appears [43]. While the quadratic form of *J* with respect to the control variable is not motivated by biological phenomena, it does ensure the optimization problem is convex (i.e., the optimization problem has several mathematical features including the existence of a global minimum).

#### 5.1.1. How Do the Weights of the Cost Functional Affect the Optimal Solutions?

**Figure 3:**
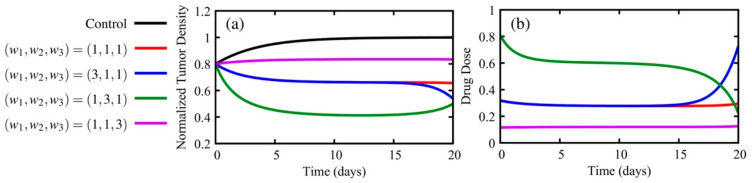
Panel (**a**) presents the normalized tumor density, while panel (**b**) displays the therapeutic dose using parameter values of *r* = 0.3 (growth rate) and *δ* = 0.45 (magnitude of the dose) for Equation (6). Depicted is the solution of the model without treatment (black) as well as the optimal control solutions for different combinations of weights. The weights depicted are: (*w*_1_, *w*_2_, *w*_3_) = (1,1,1) (red), increased weight on the tumor density at the final time (*w*_1_, *w*_2_, *w*_3_) = (3,1,1) (blue), increased weight on the tumor density over the whole treatment (*w*_1_, *w*_2_, *w*_3_) = (1, 3, 1) (green), and increased weight on the therapy toxicity (*w*_1_, *w*_2_, *w*_3_) = (1, 1, 3) (purple). By varying the weights in the optimal control functional, tumor control can vary, and the recommended drug dosage may increase or decrease over time.

To explore the consequences of different weights in the cost function, we describe the results of numerically solving the OCT problem for a simplified form of the Gompertzian growth ODE model above (see, Chapter 10 from Reference [41]):

(9) $$\frac{dT(t)}{dt}=rT(t)\ln(1/T(t))-\;\delta z(t)T(t)$$

where *r* is the growth rate, *δ* is the magnitude of the dose, and *z*(*t*) is the effect of an arbitrary therapy. The objective function is defined as:

(10) $$J(z)=w_{1}T(t_{f})+\frac{1}{2}\int_{0}^{t_{f}}(w_{2}T^{2}+w_{3}z^{2})dt$$

where the *w_i_* are the weights for each term. In Equation (10), we minimize the (1) tumor density at the final time, *t_f_* (first term on the right-hand side of the equation), (2) tumor burden during the treatment (first term in the integral), and (3) total drug dose (second term in the integral). The system was solved by implementing the Forward-Backward Sweep Method (as described in Chapter 4 of Reference [41]). For this example, the initial and final conditions were (arbitrarily) chosen as *T*(0) = 0.8 and λ(20) = *w*_1_ respectively, where the final time is 20 days and the initial normalized tumor density is 0.8. The optimal control solutions for four different weight combinations are presented in Figure 3. For comparison, if the treatment is not administered, then *T*(20) = 1, but if the weights are (*w*_1_, *w*_2_, *w*_3_) = (1,1,1), *T*(20) = 0.65. As expected, if we set *w*_3_ = 3, which weights the total drug administered as more costly, the tumor density at the final day increases to *T*(20) = 0.83. For the cases (*w*_1_, *w*_2_, *w*_3_) = (3,1,1) and (*w*_1_, *w*_2_, *w*_3_) = (1,3,1), the solutions are *T*(20) = 0.54 and *T*(20) = 0.50, respectively. Despite the fact that the tumor density at day 20 is 8% higher for the case where *w*_1_ = 3, the total dose is 46% lower than the case *w*_2_ = 3. The results from the numerical experiments presented in Figure 3 are qualitatively similar with the results obtained by Reference [111]. The definitions of the weights will significantly alter the optimal treatment protocol. In our simulations, the highest total dose when (*w*_1_, *w*_2_, *w*_3_) = (1,3,1) is 485% higher than the lowest total dose when (*w*_1_, *w*_2_, *w*_3_) = (1,1,3) (11.57 and 2.39, respectively). In practice, the definition of these weights depends upon on the specific treatment used and its potential side-effects, the type of the tumor, and the priorities for the desired treatment.

#### 5.1.2. What Clinically Relevant Constraints Might Be Considered?

Martin and Teo [86] also studied applications of OCT to chemotherapy and included additional, clinically relevant constraints to construct optimal treatment schedules. The first constraint assigned on the state variable was to account for the efficacy of the drug. As it is undesirable for the tumor burden to increase at any point during the course of therapy, the number of cancer cells, *T*(*t*), is not allowed to exceed the initial condition, *T*_0_, at any time:

(11) $$T(t)\;\leq {\;T}_{0}.$$

For a toxicity constraint, they limited the total drug exposure (calculated by integrating drug plasma concentration over the treatment interval), by imposing

(12) $$\int_{t_{0}}^{t_{f}}z\left(t\right)dt\leq z_{tot},$$

where *z_tot_* is the bound on the total drug exposure. Additionally, since the chemotherapies are not effective below certain plasma concentrations, *z*_0_, the treatment concentration is expressed in a piecewise fashion using the Heaviside function, *H*, as

(13) $$z(t)=(z(t)\;{-\;z}_{0})H(z(t)\;-\;z_{0}),$$

where

(14) $$H(z(t)\;–\;z_{0})=\left\{\begin{array}{l} {0,\;\;z(t)\;>\;z}_{0}\\ 1,\;\mathrm{otherwise} \end{array}\right..$$

Imposing the above constraints ensure the drug concentrations below *z*_0_ are ineffective but still contribute to toxicity. Also, to avoid acute toxicity, when the drug concentration exceeds a maximum *z_max_*, the control variable is constrained by the inequality

(15) $$z(t)\;\leq \;z_{max}.$$

Due to discrete dosing and the therapeutic being effective over a specific range of concentrations, the amount of drug that can be administered at any given point in time is bounded by

(16) $$z_{lb}\;\leq \;z(t_{k})\;\leq \;z_{ub}\;\mathrm{or}\;z(t_{k})=0,$$

where *z_lb_* and *z_ub_* are the lower and upper bounds of the concentration of drug respectively, given at times *t_k_*, *k* = 1, 2, …, *N*. Therefore, by defining an objective function for the number of cancer cells at *t_f_* (Equation (8)), while incorporating several constraints on the drug concentration *z*(*t*), they were able to generate regimens with clinically relevant objectives, that is, the optimal control solution satisfies constraints imposed by the established clinical knowledge regarding the drug action, such as the maximum allowed cumulative and single drug dose. In particular, they found that in contrast to conventional regimens that begin with high-intensity treatments at the beginning of therapy, an optimal drug regimen applies the largest doses at the end of the treatment period. This result is directly affected by including in the objective function the requirement that the tumor burden is minimized at the end of treatment. Also note that while this is an excellent example of applying OCT to a clinical problem, actually taking such results and translating them into a practical solution remains an open area of investigation.

#### 5.1.3. What If the OCT Problem is Dependent on More Than One Variable? What about Drug Resistance?

Two recent examples of OCT applied to PDE models with respect to chemotherapy are References [115] and [116], that discuss the clinical considerations of drug resistance. The research by Pouchol et al. [115] is a theoretical study that provides insight into the mechanisms behind the emergence of drug resistance. The authors characterize both mutations (i.e., changes in gene base pairs) and epimutations (i.e., changes in the expression of genes without a change in the sequence of DNA base pairs). In particular, the model allows for continuous transition between resistant and sensitive phenotypes *via* reversible changes in the expression of genes (epimutations). Applying OCT to this modeling framework identified the importance of “drug holidays” (i.e., periods of time free of drug) for cells to revert to a phenotypic state that is susceptible to therapy.

Later, Almeida et al. [116] sought to identify optimal chemotherapy regimens for leukemia that only incorporated spontaneous epimutations to capture induced resistance. This paper is noteworthy in that it represents an early effort attempting to compare OCT modeling results to biological data. They defined their system such that the total population, *p*(*t*), depends upon the evolution of the population density function *n*(*x*,*t*),

(17) $$p(t)=\int_{\mathbb{R}}n(x,t)dx,$$

where the number density of cells in the phenotypic state *x* over the domain ℝ (such that *x* = 0 corresponds to the highest proliferation rate in the absence of drug and *x* = 1 represents the highest level of cytotoxic drug resistance) at time *t* evolves according to

(18) $$\frac{\partial n}{\partial t}=\beta \frac{\partial^{2}n}{\partial^{2}x^{2}}+R(x,p(t)z(t))n.$$

In Equation (18), the diffusion term (first term on the right-hand side) models the effects of mutations that occur with rate β, and the reaction term (second term on the right-hand side) is defined using the functional *R* representing the fitness of the cancer cells in the phenotypic state *x* dependent upon the population size, *p*(*t*), and drug concentration, *z*(*t*). This fitness functional accounts for the net proliferation of the cancer cells in phenotypic state *x* using terms defining growth, natural death, and cell kill due to chemotherapy [116]. Simply exploring the model system, the authors found several clinically relevant results including how: (1) periodic piecewise-constant delivery (which accounts for many standard regimens) can induce a population bottleneck inducing drug resistance, (2) high drug doses may effectively reduce cell number at the cost of inducing greater cell resistance, and (3) regimens that have longer drug-free periods (drug holidays) may allow cells in resistance phenotypic states to revert to more drug-sensitive states—the same result presented in Reference [115]. With models that consider the adaptability (or plasticity) of tumor cells to revert to a phenotype that is once again susceptible to therapy after treatment has been withheld, OCT can be used to potentially overcome drug resistance by generating regimens that exploit this reversible evolvability.

For the OCT problem, the authors considered one scenario aimed at minimizing the average number of cancer cells over the whole course of treatment and a second scenario aimed at minimizing the number of cancer cells at the final time: i.e., similar to above (Equation (10)) with *J* = $\frac{1}{T}\int_{0}^{t_{f}}p$(*t*)*dt* and *J* = *p*(*t_f_*), respectively. Additionally, they incorporated constraints that account for maximum tolerated dosages over the course of therapy:

(19) $$\overset{k+M_{k}}{\underset{j=k}{\sum }}z_{j}\;\leq {\;C}_{k}$$

where *z_i_* represents the *jth* dose, *C_k_*, is the maximum administrable dose for the *k^th^* therapy cycle, and *M_k_* is the maximum duration of therapy. By solving the optimal control problem, the authors identified two major conclusions. First, they found that if the goal is to minimize the number of cancer cells at the final time point, the optimal dosing regimens consists of high doses toward the end of the treatment period. Note that this is a similar result to the Gompertzian example described above that considered the tumor at the final time only. However, Almeida et al. acknowledge that such a regimen is not ideal for treatment, and by running comparison simulations, if the goal is to simply minimize the tumor burden only at the final time, standard regimens may be sufficient. Second, if the goal is to minimize the average cancer cell population size throughout the course of therapy, a continuous low dose over the therapy period has relatively the same performance compared to the optimal result (which consisted of higher doses separated by 4–7 days early in treatment followed by low daily doses). This low-dose regimen is also known as metronomic dosing and has been previously explored clinically to help minimize side effects for patients [117].

### 5.2. Examples for Radiation Therapy

Hahnfeldt et al. developed a model to describe tumor development in a murine model of lung cancer [118]. In their model, tumor growth is assumed to be Gompertzian, with the carrying capacity being proportional to the vascular support (i.e., tumor growth due to nutrients provided by the vasculature):

(20) $$\frac{dT}{dt}=-rT\ln\left(\frac{T}{K}\right),$$

(21) $$\frac{dK}{dt}=S(T,K)\;-\;I(T,K),$$

where *T* is the number of tumor cells, *K* is the effective vascular support provided to the tumor, and *r* is the tumor cell proliferation rate. The vascular development is modeled as the balance between stimulatory, *S*(*T*,*K*), and inhibitory effects, *I*(*T*,*K*):

(22) $$S(T,K)=bK^{2/3},$$

(23) $$I(T,K)=dK^{4/3},$$

where *b* and *d* are the birth and death rates of the cells, respectively. The model was extended to account for the effects of anti-angiogenic drugs on endothelial cells as well as radiotherapy [96] on both the tumor and endothelial cells:

(24) $$\left\{\begin{array}{c} \frac{dT}{dt}=-r\mathrm{Tln}\left(\frac{T}{K}\right)-\overset{\overset{\mathrm{radiotherapy}}{⏞}}{DT(\alpha_{T}+\beta_{T}\int_{0}^{t_{f}}D(s)\exp(-\mu_{T}\left(t\;-\;s))ds\right)}\\ \frac{dK}{dt}=-\gamma K\nu +S(T,K)\;-\;I(T,K)\;-\;\overset{\overset{\mathrm{radiotherapy}}{⏞}}{DK(\alpha_{K}+\beta_{K}\int_{0}^{t_{f}}D(s)\exp(-\mu_{K}\left(t\;-\;s))ds\right)} \end{array}\right.,$$

where *D* is the radiation dose, *α_i_* are the parameters from the linear component of the radiotherapy, *β_i_* are the parameters from the quadratic component of the radiotherapy, the *μ_i_* are the repair rates of the DNA breaks, *v* is the dose of the anti-angiogenic drug, and γ is treatment efficacy.

#### 5.2.1. How Do Effects of Therapy on Both Healthy and Tumor Cells Affect the OCT Problem?

The objective of their optimal control problem is to maximize the tumor cure probability (TCP), while constraining the total dose of the anti-angiogenic and the radiation therapies. The objective function is defined as *J* = max(TCP), where the TCP is given as TCP = exp(−*F*·*θ*·*T_f_* ), where *F* is the fraction of tumor cells that are capable of regeneration, *θ* is the tumor cell density, and *T_f_* is the tumor volume at the end of the treatment. To be clinically relevant, the radiotherapy must be given at fixed intervals. This constraint is enforced in their optimal control problem by requiring the radiotherapy to be administered for one minute per day but that each daily dose can be different. For the case where the anti-angiogenic drug is not considered, the resulting optimal radiotherapy protocol is to administer an increasing dose of radiation with each session, where the final dose is double the initial dose. However, when the anti-angiogenic drug is included with the radiotherapy in the optimal control problem, the optimal regimen for the radiation treatment is to initially decrease the dose until the midpoint of treatment, reaching half of its initial dose value, and then increasing the dose until the end of treatment, reaching the same initial fraction as at the start of therapy. Further, among all possible strategies (e.g., sequential versus simultaneous versus partial overlap versus alternating, dose intensification versus constant dose rate versus dose de-escalation), their results indicate that the optimal scheme is to administer the anti-angiogenic drug at the latter portion of the radiotherapy treatment in a dose-intensified manner. This optimal scheme is consistent with the results in Reference [119], where the authors concluded that the simultaneous treatment is more effective in reducing the tumor size in mice than radiotherapy followed by an anti-angiogenic drug.

#### 5.2.2. How Do We Account for the Spatial Distributions of Radiation Dose on Normal Tissue?

An example of applying OCT to a reaction-diffusion model is provided by Corwin et al. [78,79], who worked to optimize intensity-modulated radiation therapy (i.e., spatially varying radiation) for glioblastoma patients. To characterize relevant clinical objectives, they considered two equally important decision criteria: (1) minimizing radiation dose to normal tissue, and (2) minimizing the number of viable tumor cells after seven days. To provide dose fractions that are comparable to the standard of care, they incorporated several restrictions including limiting the total daily dose to the normal tissue and ensuring that the fraction of dose remains well below a threshold (interpreted as a tolerance threshold). Due to a spatially varying control variable, the domain is discretized into imaging voxels, and a quadratic objective functional (again, where the quadratic ensures the convexity of the optimization problem for a global minimum) is considered with the general form,

(25) $$J=\sum_{i}\sum_{j}W^{i}{\left(D^{i}\;–\;z^{i,j}\right)}^{2}H(D^{i}\;–\;z^{i,j}),$$

where *z^i,j^* is the dose received by the *j*th voxel of the *i*th decision criterion (the different decision criteria are described below), *W^i^* is the weight of the *i*th criterion, *D^i^* is the dose parameter that describes the cost of the *i*th decision criteria, and *H*$\left(D^{i}\;-\;z^{i,j}\right)$ is the Heaviside function that sets the weight to 0 in voxels with *D^i^* > *z^i,j^* and is 1 otherwise. The authors chose an alternative strategy for solving their optimal control problem, where instead of incorporating decision criteria directly into the objective functional (i.e., Equation (25)), the authors employed a multi-objective evolutionary algorithm (for more information see Reference [79]) to optimize the weights and dose. The quality of each optimized plan was judged by three decision criteria: (1) and (2) being minimizing the maximum dose per fraction to any voxel and minimizing the uniform equivalent dose to normal tissue, respectively. A third decision criterion was also varied to investigate the effects of altering target optimization objectives: (3a) tumor cell kill, (3b) tumor survival after one week of treatment, or (3c) tumor survival 11 weeks after one week of treatment. Solving this optimization problem, they generated spatially heterogenous radiation fields that, when compared to the standard-of-care plan, resulted in an overall decrease in the equivalent uniform dose delivered to normal tissue while simultaneously increasing the dose to tumor tissue by almost a factor of three for some patients.

## 6. Challenges and Opportunities

A fundamental barrier to the practical and productive application of OCT to optimize the response of a tumor to therapy is our current limits on being able to accurately predict—mathematically—the growth of the individual tumor, the distribution of drugs within that tumor, and the response of that tumor’s cells have to various therapies. The development of multiscale mathematical models to incorporate several levels of biological data for an individual patient is another area for future investigation [120]. The computational challenges of implementing such models as well as methods for solving the optimal control problem for these systems are also active research areas. Finally, we are currently limited in our ability to adequately quantify the *in vivo* distribution of therapies, though there are new efforts underway in this area [48,121,122].

**Figure 4:**
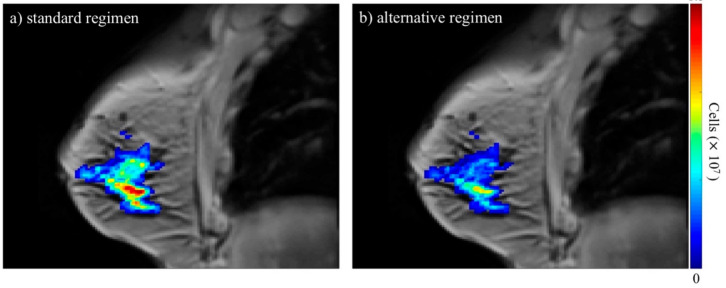
The figure depicts anatomical images of a central slice of the breast overlaid with the predicted total tumor cellularity in color. Panel (**a**) is the model-predicted tumor response for the standard-of-care regimen the patient actually received while panel (**b**) is the predicted response for a regimen of the same total dose with alternate dosing. This simulation indicates that the tumor burden may have been better controlled using the alternative regimen. These results were achieved by using a mathematical model [130] that is calibrated using quantitative magnetic resonance imaging (MRI) data prior to and after one cycle of therapy to define patient-specific parameter values. After calibration, the model is then simulated forward to the time of completion of therapy for the standard regimen and an alternative regimen for comparison. The standard-of-care regimen consisted of combination doxorubicin and cyclophosphamide every two weeks and the alternate regimen pictured consisted of a 1/14 of a dose administered daily. Studies such as these provide key motivation for the potential of applying OCT to the problem of optimizing therapeutic regimens on a patient-specific basis.

While there is a great deal of data collected from patients undergoing treatment for cancer, not all of it is well suited or easily adapted for biologically based modeling efforts [64,65]. There are several data types routinely collected from patients (e.g., medical imaging and tissue samples), but challenges arise due to the wide variation currently employed in obtaining these data. For example, routine imaging data (e.g., magnetic resonance imaging (MRI) or positron emission tomography (PET)) might be collected in most patients, but they are frequently not of the kind that can be used for quantitative modeling—rather they are collected merely for anatomical evaluation (as in, for example, applying the response evaluation criteria in solid tumors (RECIST) [123]). This is especially manifest during multi-site clinical trials in which different imaging devices are used to collect the data. Fortunately, there are ongoing efforts designed to establish consensus including the National Cancer Institute’s Quantitative Imaging Network [124], which has worked to establish the repeatability and reproducibility of advanced imaging techniques [125,126,127,128]. There has recently been an increase in use of medical imaging data to inform patient-specific models of tumor growth and response [129]. In principle, images can be collected early in the course of therapy and these data can be used to calibrate an appropriate mathematical model to determine patient-specific parameters of tumor response to that therapeutic regimen. Using this patient-specific information, an optimal control problem can be solved to suggest improvements in the dosing strategy for the individual patient—see Figure 4 for an example of the predicted spatio-temporal development of a breast cancer tumor for a standard-of-care regimen compared to an alternative dosing regimen using mathematical modeling [130]. Emerging MRI and PET imaging methods can provide non-invasive characterization of properties including (for example) cellularity, perfusion, permeability, hypoxia, metabolism, and proliferation [131,132,133,134,135,136,137]. As changes in these properties are often temporally upstream of volumetric changes, such data can be used to determine patient-specific response parameters long-before the conclusion of treatment. In principle, calibrating a mathematical model with patient-specific, three-dimensional (3D) imaging data can provide a predictive framework appropriate for applying the methods of OCT to determine the best dosing strategy for the individual patient.

An additional challenge that must be addressed is the uniform processing of data. Beyond the challenges mentioned above pertaining to the collection of data, the current methods for processing data must be standardized. While the general procedures for assessing clinical data may be the same between institutions, the results can vary between individuals because many of these methods are not quantitative, allowing for bias and human error to permeate analysis and interpretation of results. For example, in radiology, it has been well documented that various factors can cause errors and discrepancies between experts in the reading of images [138]. More broadly, there exists a repeatability and reproducibility crisis in cancer research (i.e., The Reproducibility Project [139]), and this in part can be attributed to the inconsistent processing of data. Robust and precise methods of data processing must be developed to not only provide consistent results for any patient, experiment, or dataset, but also to alleviate the burden of “hand processing” data.

Uncertainty quantification, propagation, and mitigation are also central for the proper application of OCT. Optimal control is a decision-making process and should account for and/or address the uncertainties in its results. Therefore, an important problem in applying OCT to cancer treatment is to characterize all sources of uncertainties, trace their propagation through the various analysis steps, quantify the uncertainty in model prediction, and ultimately mitigate these uncertainties through the control decision making process. Models inherently have uncertainty in their construction—a term known as model inadequacy. Nevertheless, if a model can be built or modified to be constrained and initialized with measurable data—to produce predictions that are directly comparable to clinical or experimental results—these uncertainties can begin to be understood and considered as part of the model’s predictions. However, even high-quality measurements still have noise and variability between subjects (data uncertainties) that can propagate through model simulations. Solutions of optimal control problems depend on the uncertainty in mathematical models and the data of tumor development. Robust optimal control problems should address uncertainties in their formulations. There are several mathematical and computational tools to aid in assessing uncertainties in model prediction. Parameter sensitivity analysis is a broad group of uncertainty assessment techniques used to identify and rank parameters by their relative importance (i.e., those parameters that cause the greatest variation in the outputs of interest [140,141]). Other methods for handling uncertainty are Bayesian model calibration and validation, as it provides a framework for identifying the essential features of a predictive model while also providing means of assessing both data uncertainty and model inadequacy [75]. Based on contemporary treatments of statistical inverse analysis, in the Bayesian approach, the model parameters are random variables or processes characterized by probability theory. Efficient computational methods for solving models with random parameters have emerged in recent years [142,143,144], and the development of these analyses of control formulations within a probabilistic setting are ongoing [145,146,147,148].

With the development of various modalities of cancer treatment, we are presented with a unique opportunity to not only optimize these regimens individually but also in combination. Currently, many combination therapies are given at the same time (such as cytotoxic therapies with targeted and/or immune therapies) or consecutively (such as surgery followed by radiation). As an example, with our growing understanding of the immune system and the various ways white blood cells can be manipulated (check point inhibitors, adoptive cell transfer, cytokine and antibody injections, and even vaccines), the order, timing, and dosage of different immunotherapies may be optimized with radiation and chemotherapy [149,150,151,152]. Many of the current applications of OCT are formulated as continuous control problems, and more studies of discontinuous dosing should be explored. Furthermore, and of critical importance, other potential regimens not yet considered could be systematically evaluated. Such regimens may drive new technology for the administration of drugs. For example, take-home infusion pumps [153] may be further developed and made more widely available, allowing drugs to be administered over longer periods (from hours to days) giving a lower dose over time to decrease potential side effects while also treating the cancer continuously.

## 7. Conclusions

We have presented an overview of the components of OCT that are necessary for optimizing therapeutic regimens for oncology patients. While several challenges need to be addressed, it is clear that this represents a new and exciting possibility within the broader field of mathematical oncology. It is our hope that this contribution raises awareness of OCT for oncology, so that the next era of cancer treatment protocols will be shaped by model forecasts that are optimized for the individual patients using rigorous mathematical methods.

## Supplementary Materials

The Supplementary Materials provide descriptions of Pontryagin minimization principle along with an introductory example of its application for the more interested reader available online at https://www.mdpi.com/2077-0383/9/5/1314/s1.
