# Supplementary material for: Optimal Control Theory for Personalized Therapeutic Regimens in Oncology: Background, History, Challenges, and Opportunities
Source: J Clin Med. 2020 May 2;9(5):1314. doi: 10.3390/jcm9051314 (PMC7290915; doi:10.3390/jcm9051314)
Supplement: Supplementary file 1 [file jcm-09-01314-s001.pdf]

1.1. Pontryagin Minimum Principle and Optimality Conditions

Following the method of Lagrange [1], a control Hamiltonian function H can be constructed by appending the state equation to the integrand L using the Lagrange multipliers, $\lambda(t)$, as follows:

$$H(u(t), z(t), \lambda(t), t) = L(u(t), z(t), t) + \lambda^T(t) f(u(t), z(t), t), \tag{1}$$

where $z(t)$ is the optimal control, and $u(t)$ is the corresponding optimal state. Then the Pontryagin Minimum Principle (PMP; [1]) states that there exists a continuous function λ, known as an *adjoint function*, that is the solution of the *adjoint equation*

$$\dot{\lambda}(t) = -H_u(u(t), z(t), \lambda(t), t), \tag{2}$$

along with the appropriate initial (or final) condition of λ. In Eq. (2), H_u denotes the differentiation of the Hamiltonian function with respect to the state. In particular, the adjoint function is a Lagrange multiplier that brings the information of the state equation constraint to the optimization problem. According to the PMP, the optimal control, $z(t)$, and corresponding optimal state, $u(t)$, and adjoint, $\lambda(t)$, must minimize the Hamiltonian so that

$$H(u(t), z(t), \lambda(t), t) \leq H\big(u(t), z^*(t), \lambda(t), t\big), \tag{3}$$

for all time and for all admissible (i.e., feasible) trajectory control variables $z^*(t)$, while the adjoint equation Eq. (2) is satisfied. Admissible trajectories are defined as a set of variables that lay in the neighborhood of the minimal solution and satisfies all of the constraints.

With the above considerations, we can now define the necessary and sufficient conditions for optimality. For a feasible trajectory that satisfies the minimum principle, condition Eq. (3) implies that the Hamiltonian is minimum at the optimal control $z(t)$, such that

$$H_z(u(t), z(t), \lambda(t), t) = 0. \tag{4}$$

The condition (4) is the *first order necessary condition* for optimality and corresponds to a special case of the Euler-Lagrange equation of the calculus of variations. The Pontryagin Minimum Principle also leads to the positive semi-definiteness of the Hamiltonian's Hessian matrix as

$$H_{zz}(u(t), z(t), \lambda(t), t) \geq 0, \tag{5}$$

that is termed the Legendre-Clebsch condition and it is a *second-order necessary condition* for optimality. In addition to the necessary conditions derived from the Pontryagin Minimum Principle, if the strengthened Legendre-Clebsch condition

$$H_{zz}(u(t), z(t), \lambda(t), t) > 0, \tag{6}$$

holds, it guarantees that $z(t)$ is the local minimizer of the Hessian. The condition in Eq. (6) is known as the *second-order sufficient condition* for local optimality. For a purely convex objective functional with respect to the control variables, the necessary conditions are sufficient to guarantee the optimal control variable. We refer the interested readers to [1, 2] for the proof of these theorems.

In summary, the Pontryagin Minimum Principle converts the optimal control problem into a multi-point boundary value problem. That is, the optimality condition $H_z = 0$ results in control expressed as,

$$z(t) = G(u(t), \lambda(t), t), \tag{7}$$

and the optimal control variable and corresponding state and adjoint can be computed by solving an ODE system,

$$\dot{u}(t) = f\big(u(t), G(u(t), \lambda(t), t)\big), \tag{8}$$

$$\dot{\lambda}(t) = -H_u\big(u(t), G(u(t), \lambda(t), t)\big), \tag{9}$$

with appropriate initial and end time conditions, while (6) ensures the optimal control is a minimizer. A specific example of the methods described in this section is presented in section 1.4.

1.2 One example using the Pontryagin Minimum Principle

Here we outline the steps for using the Pontryagin Minimum Principle (PMP) to optimize treatment delivery, given a tumor growth model (see, Chapter 10 from [11]), and we explore the effects of different weights in the objective function. The text below represents a specific case of the general formulation offered in section 1.1.

Begin by considering the following ODE model that describes the temporal evolution of tumor density, T:

$$\frac{dT}{dt} = rT\ln(1/T) - \delta z(t)T, \tag{10}$$

where r is the growth rate, δ is the magnitude of the dose, and $z(t)$ is the effect of an arbitrary therapy (i.e., systemic or radiation therapy). Eq. (10) represents a specific example of Eq. (1) from the main text. For this example, the objective function (see Eq. (2) in the main text) is defined as:

$$J(z) = w_1 T(t_f) + \frac{1}{2}\int_0^{t_f}\left(w_2 T^2 + w_3 z^2\right)dt, \tag{11}$$

where the w_i are the weights for each term. In Eq. (11), we minimize the 1) tumor density at the final time, t_f (first term on the right-hand side of the equation), 2) tumor burden during the treatment (first term in the integral), and 3) total drug dose (second term in the integral). Following the method of Lagrange, the solution of the optimal control problem is obtained by first introducing the Hamiltonian, H, given by:

$$H(\mathrm{u}(t), z(t), \lambda(t), t) = L(\mathrm{u}(t), z(t), t) + \lambda^{\mathrm{T}}(t)\, f(\mathrm{u}(t), z(t), t), \tag{12}$$

where the Hamiltonian function, H, is constructed by appending the state equation (Eq. (10)) to the integrand L using Lagrange multipliers, $\lambda(t)$. Therefore, according to the PMP, the Hamiltonian for this example becomes:

$$H(t, T, z, \lambda) = \frac{w_2}{2}T^2 + \frac{w_3}{2}z^2 + \lambda rT\ln(1/T) - \lambda\delta zT, \tag{13}$$

then the PMP states that there exists a continuous function λ, known as the *adjoint function*, that is the solution of the *adjoint equation*:

$$\frac{d\lambda}{dt} = -H_u(u(t), z(t), \lambda(t), t), \tag{14}$$

along with the appropriate initial (or final) condition of λ. In Eq. (14), H_u denotes the differentiation of the Hamiltonian function with respect to the state variable. The adjoint function is a specific Lagrange multiplier that brings the information of the state equation constraint into the optimization problem. For our example, the adjoint is computed by a solution of the adjoint ODE:

$$\frac{d\lambda}{dt} = -w_2 T - \lambda(\ln(1/T) - 1) + \lambda\delta z). \tag{15}$$

According to the PMP, the optimal control, $z(t)$, and corresponding optimal state, $u(t)$, and adjoint, $\lambda(t)$, must minimize the Hamiltonian such that

$$H(u(t), z(t), \lambda(t), t) \leq H(u(t), z^*(t), \lambda(t), t), \quad (16)$$

for all time and for all admissible control variables, $z^*(t)$, while satisfying the adjoint Eq. (14). Admissible (i.e., feasible) control variables are defined as the set of trajectories that lay in the neighborhood of the minima and satisfy all of the constraints.

With these considerations, we can now define the necessary and sufficient conditions for optimality. Considering feasible trajectories for the optimal control problem that satisfy the PMP, the condition summarized by Eq. (16) indicates that the Hamiltonian is a minimum for the optimal control $z(t)$ when

$$H_z(u(t), z(t), \lambda(t), t) = 0. \quad (17)$$

The condition from Eq. (17) is the *first order necessary condition* for optimality and corresponds to a special case of the Euler-Lagrange equation. The PMP also requires the positive semi-definiteness of the Hamiltonian's Hessian matrix:

$$H_{zz}(u(t), z(t), \lambda(t), t) \geq 0. \quad (18)$$

Eq. (18) is termed the Legendre-Clebsch condition, and it is *the second-order necessary condition* for optimality. In addition to the necessary conditions derived from the PMP, if the strengthened Legendre-Clebsch condition

$$H_{zz}(u(t), z(t), \lambda(t), t) > 0 \quad (19)$$

holds, it guarantees that $z(t)$ is a local minimizer of the Hessian. The condition of Eq. (19) is known as the *second-order sufficient condition* for local optimality. For a convex objective (i.e., there is one optimal solution) with respect to the control variables, the necessary conditions are sufficient to guarantee an optimal control solution. (We refer the interested readers to [36, 37] for the proof of these theorems.) Given the Hamiltonian in Eq. (13), the optimality conditions for our example are:

$$\begin{cases} H_z = 0 \\ w_3 z - \lambda\delta T = 0 \\ z = \dfrac{1}{w_3}\lambda\delta T \end{cases}. \quad (29)$$

Ultimately, the PMP converts the optimal control problem into a multi-point boundary value problem. That is, the optimality condition, $H_z = 0$, results in control solutions expressed as a function of the state and adjoint functions, and the optimal control variable and corresponding state and adjoint can be computed by solving:

$$\begin{cases} \dfrac{\partial \boldsymbol{u}}{\partial t} = f(u(t), z(u(t), \lambda(t)), t) \\ \dfrac{d\boldsymbol{\lambda}}{dt} = -H_u(u(t), z(t), \lambda(t), t)' \\ z(t) = G(u(t), \lambda(t)) \end{cases} \quad (30)$$

where $\boldsymbol{G}$ is a function for which $\boldsymbol{z}$ is described. With appropriate initial and end time conditions, Eq. (28) ensures the optimal control is a minimizer. Thus, for our tumor density example, to find the optimal solution, we need to solve the following system

$$\begin{cases} \dfrac{dT}{dt} = rT\ln(1/T) - \delta z T \\ \dfrac{d\lambda}{dt} = -w_2 T - \lambda(\ln(1/T) - 1) + \lambda\delta z. \\ z = \dfrac{1}{w_3}\lambda\delta T \end{cases} \quad (31)$$

See the main text subsection 5.1 and Figure 2 for example optimal solutions for this system and the results of exploring different weights within the cost functional.

1. Kirk, D.E., *Optimal control theory: an introduction*. 2012: Courier Corporation.

2. Lee, E.B. and L. Markus, *Foundations of optimal control theory*. 1967: John Wiley & Sons, Inc.
3. Akkouche, A., A. Maidi, and M. Aidene, *Optimal control of partial differential equations based on the Variational Iteration Method.* Computers & Mathematics with Applications, 2014. **68**(5): p. 622-631.
4. Ramirez, W.F., *Process control and identification*. 1994: Academic Press.
5. Barzilai, J. and J.M. Borwein, *Two-point step size gradient methods.* IMA journal of numerical analysis, 1988. **8**(1): p. 141-8.
6. Yuan, Y.-x., *Step-sizes for the gradient method.* AMS IP Studies in Advanced Mathematics, 2008. **42**(2): p. 785.
7. Attouch, H., J. Bolte, and B.F. Svaiter, *Convergence of descent methods for semi-algebraic and tame problems: proximal algorithms, forward-backward splitting, and regularized Gauss-Seidel methods.* Mathematical Programming, 2013. **137**(1-2): p. 91-129.
8. Combettes, P.L. and J.C. Pesquet, *Proximal Splitting Methods in Signal Processing.* Fixed-Point Algorithms for Inverse Problems in Science and Engineering, 2011. **49**: p. 185-212.
9. Betts, J.T., *Practical methods for optimal control and estimation using nonlinear programming.* Vol. 19. 2010: SIAM.
10. Kelley, C.T., *Iterative methods for optimization*. 1999: SIAM.
11. Lenhart, S. and J.T. Workman, *Optimal control applied to biological models*. 2007: Chapman and Hall/CRC.
